# Revealing the Formation Mechanism of Key Metabolites During Japonica Rice Storage Driven by Microbial Functional Genes

**DOI:** 10.3390/metabo16050302

**Published:** 2026-04-29

**Authors:** Xinwei Li, Wei Deng, Zongrui Zhang, Hui Tong, Yi Cao

**Affiliations:** 1 Liaoning Academy of Agricultural Sciences, Liaoning Grain Science Research Institute, Shenyang 110161, China; xinweili.love@163.com (X.L.);; 2 Tianjin Agricultural University, Tianjin 300384, China

**Keywords:** aromatic japonica rice, storage, metabolomics, metagenomics, differential metabolites, fungal community

## Abstract

Background: To elucidate the evolution of metabolites and fungal communities during storage of fragrant japonica rice (Liaoxiangjing 1396), and to investigate the biosynthetic mechanisms of key compounds and their association with quality deterioration, this study examined rice samples stored under simulated conditions for 16 months. Method: Samples were collected at 4-month intervals (designated R20, R14, R13, R12, and R11). Metabolites were identified using GC-MS non-targeted metabolomics, while fungal community structure was analyzed through metagenomics. Core mechanisms were further elucidated via PLS-DA, KEGG pathway enrichment, and multiomics association analysis. Result: Results demonstrated that the fatty acid content of rice increased initially and then stabilized (from 12.24 mg/g in R20 to 17.63 mg/g in R12). A total of 263 metabolites were identified, with oxygenated organic compounds (38 species) and lipids/lepidid molecules (24 species) as the predominant categories. Twelve key differential metabolites were screened from the R20 and R12 groups, involving five major metabolic pathways, including amino acid metabolism and lipid metabolism. In the fungal community, *Pseudomonas* (60.2%) and *Pantoea* (38.19%) were dominant taxa, with a specific *Pantoea* species (*Pantoea* sp.) identified as a core potential biomarker. Multiomics association analysis revealed that *Klebsiella* dominated the ndhB energy metabolism pathway, while multiple bacteria cooperatively regulated the mcp chemotaxis pathway, interacting with monosaccharide and amino acid accumulation. Conclusions: This study reveals that the storage quality deterioration of fragrant japonica rice is driven by the “metabolite–microbe-pathway” chain regulation, and the dynamic changes in key metabolites and fungal communities can serve as quality early warning targets.

## 1. Introduction

Japonica rice, prized for its distinctive flavor and superior palatability, plays a pivotal role in the rice industry [1,2]. Its quality stability during storage directly impacts market value and consumer experience [3]. During storage, environmental factors like temperature and humidity trigger physiological and biochemical changes—lipid oxidation, starch degradation, and protein hydrolysis—which elevate fatty acid levels, diminish flavor, and degrade taste [4,5,6]. In recent years, multiomics technologies have become essential tools for deciphering grain storage quality evolution. Metabolomics precisely tracks dynamic changes in quality-related metabolites, while metagenomics reveals microbial community regulation of quality [7]. Their integrated application provides novel insights into the “metabolism–microbiota-quality” interplay.

Current research on rice storage primarily focuses on single indicators or single-omics analyses, such as the correlation between fatty acid values and quality [8,9] or the variation patterns of specific metabolites [10,11]. However, systematic elucidation remains lacking regarding the evolutionary pathways of key compounds, biological formation mechanisms, and interactions with fungal communities during the storage of aromatic japonica rice. Existing studies indicate that metabolic differences in high-quality rice during storage mainly concentrate on carbohydrate, lipid, and amino acid metabolic pathways [11,12], while structural changes in microbial communities may accelerate or delay quality deterioration by regulating metabolic pathways. Nevertheless, the variation patterns of characteristic flavor compounds (e.g., terpenes and esters) in aromatic japonica rice during storage [13,14], as well as the regulatory mechanisms of core functional microorganisms on key metabolites, remain unclear. This limitation hinders the precise development of storage quality control technologies.

Liaoxiangjing 1396, a high-quality aromatic japonica rice variety developed by the Liaoning Provincial Rice Research Institute, holds significant practical value in the study of storage quality stability. This research systematically analyzed the composition of metabolites, fungal community structure, and core metabolic pathways of rice grains at different storage stages by simulating real-world storage conditions and integrating non-targeted metabolomics and metagenomics technologies. It identified key differential metabolites and potential biomarkers related to quality, elucidated the biosynthetic mechanisms of critical compounds, and provided theoretical support and technical references for the precise regulation and early warning technology development of storage quality in aromatic japonica rice.

## 2. Materials and Methods

### 2.1. Sample Collection

Using Liaoxiangjing 1396 rice (collected in October 2023) as the study subject, which was cultivated by the Liaoning Provincial Rice Research Institute, moisture content was measured post-harvest using a moisture analyzer. The rice grains exhibited a moisture content of 21.2–24.5% with impurities ≤ 0.3%, and were transferred to a simulated storage facility for 16 months. Upon harvest and prior to storage, the rice grains exhibited a natural initial moisture content ranging from 21.2% to 24.5% (measured across the bulk sample), with impurities ≤ 0.3%. No artificial moisture adjustment was applied before the start of the simulated storage period. The experiment utilized a simulated storage bin measuring 1.1 m in length and width, with a height of 1.8 m. Through ventilation control, storage conditions were adjusted to: temperature 17–25 °C, relative humidity 45–75%. To ensure precise control and reproducibility, the temperature and relative humidity within the simulated storage bin were continuously monitored using high-precision digital temperature and humidity sensors. Specifically, data were recorded three times daily (in the morning, afternoon, and evening). The daily average values were then calculated from these three readings to represent the environmental conditions for that day. The grain pile height was set at 1.2 m, and simulated storage was conducted according to actual warehouse standards. Samples were collected every 4 months. The sampling stages were labeled as: R20 (initial control, 0 months), R14 (4 months), R13 (8 months), R12 (12 months), and R11 (16 months). At each interval, a 150 g sample was collected. The samples were divided into 3 parallel aliquots (50 g each) and stored in sterile homogenization bags at 4 °C in a laboratory environment for subsequent use.

### 2.2. Experimental Methods

#### 2.2.1. Sample Processing

Pulverize and grind the rice through a 0.2 mm sieve to obtain a powdered sample. Accurately weigh 50 mg of the sample into a 2 mL grinding tube, add 0.5 mL of methanol-water solution (CH_3_OH:H_2_O *V*:*V* = 4:1, containing 0.02 mg/mL of the internal standard L-2-chloro-L-phenylalanine), then add a steel bead and 200 μL of chloroform. Grind it in a 20 °C grinder (50 Hz, 3 min) twice. Perform low-temperature ultrasonic extraction for 30 min at −20 °C, followed by 30 min at −20 °C. Low-temperature centrifuge for 15 min (13,000 rcf, 4 °C). Collect the supernatant and transfer it to a glass derivatization bottle, then dry under nitrogen. Add 80 μL of 15 mg/mL pyridine hydrochloride of 8-mercaptopurine to the derivatization bottle, vortex for 2 min, then incubate in a shaker at 37 °C for 90 min to complete the oximation reaction. After removing the sample, add 80 μL of BSTFA (containing 1% TMCS) as the derivatization reagent, vortex for 2 min, then react at 70 °C for 60 min. Remove the sample, let it stand at room temperature for 30 min, and proceed with omics analysis.

#### 2.2.2. Fatty Acid Value

The fatty acid value was determined according to the national standard of China GB/T 20569-2006 [15]. The fatty acid value (FAV) was determined following the Chinese National Standard GB/T 20569-2006. Briefly, rice samples were ground and passed through a 40-mesh sieve. A 10 g sample was extracted with 50 mL of anhydrous ethanol by shaking for 10 min. The extract was filtered, and 25 mL of the filtrate was titrated with 0.01 mol/L potassium hydroxide (KOH) standard solution using phenolphthalein as an indicator until a faint pink color persisted for 30 s. FAV was calculated as milligrams of KOH required to neutralize free fatty acids in 100 g of dry sample and expressed as mg KOH/100 g.

#### 2.2.3. Sample Genome DNA Extraction and PCR Amplification

Sample DNA was extracted using the FastPure Stool DNA Isolation Kit (Magnetic bead) (MJYH, Shanghai, China). After DNA extraction, the DNA concentration and purity were detected, and DNA integrity was assessed by 1% agarose gel electrophoresis. DNA was fragmented using Covaris M220 (Genentech, Shanghai, China) to screen for fragments approximately 350 bp in length, which were used to construct a PE library. The standard fungal ITS1 fragment length is 250 bp, with the upstream primer being ITS5F (GGAAGTAAAAGTCGTAACAAGG) and the downstream primer being ITS1R (GCTGCGTTCTTCATCGATGC).

#### 2.2.4. PE Library Construction and Metagenomic Sequencing

Magnetic beads were used to screen and remove self-joining fragments of the adapter, followed by PCR amplification for library template enrichment. The PCR products were recovered using magnetic beads, and the library was constructed with NEXTFLEX Rapid DNA-Seq (Bioo Scientific, Austin, TX, USA) to obtain the final library. Metagenomic sequencing was performed on the Illumina NovaSeq™ X Plus (San Diego, CA, USA) platform (Shanghai Meiji Biomedical Technology Co., Ltd. Shanghai, China).

#### 2.2.5. GC-MS Detection

Chromatographic conditions: The derivatized sample was injected into the GC-MS system in split mode for analysis, with an injection volume of 1 µL and a split ratio of 15:1. The sample was separated on a DB-5MS capillary column (40 m × 0.25 mm × 0.25 µm, Agilent 122-5532G, Agilent Technologies, Santa Clara, CA, USA) before entering the mass spectrometry detection. The injection port temperature was 260 °C, with high-purity helium as the carrier gas at a flow rate of 1 mL/min, a purge flow rate of 3 mL/min, and a solvent delay of 5.5 min. The heating program was as follows: initial temperature of 60 °C, equilibrium for 0.5 min, followed by a ramp to 310 °C at a rate of 8 °C/min, maintained for 6 min.

Mass spectrometry conditions: Electron Impact Ionization (EI), transmission line temperature 310 °C, ion source temperature 230 °C, quadrupole temperature 150 °C, electron energy 70 eV. Scanning mode: Full scan (SCAN), mass range: *m*/*z* 50–500, scan frequency: 3.2 scans/s. The total run time for a single sample injection in our GC-MS method was 40 min.

### 2.3. Data Analysis

Principal component analysis (PCA) and orthogonal partial least squares discriminant analysis (OPLS-DA) were performed on the preprocessed data matrix using the ropls package (Version 1.6.2) in R, with model stability evaluated through 7-fold cross-validation. To evaluate the statistical significance of differences in metabolite abundances across different storage stages, one-way analysis of variance (ANOVA) was performed, followed by Tukey’s honestly significant difference (HSD) post hoc test for multiple comparisons [16]. The selection of significantly different metabolites was based on variable importance scores (VIP) derived from the OPLS-DA model and the *p*-value of Student’s *t*-test, with metabolites defined as significantly different when VIP > 1 and *p* < 0.05. Pathway annotation of the differentially expressed metabolites was conducted using the KEGG database (https://www.kegg.jp/kegg/pathway.html, accessed on 19 April 2025) to identify the metabolic pathways involved. Pathway enrichment analysis was performed using the scipy.stats package in Python (version 3.9.7, Python Software Foundation, Wilmington, DE, USA), with Fisher’s exact test employed to determine the most biologically relevant pathways to experimental treatments [17]. Non-redundant gene sets were aligned with the KEGG database (using BLASTP (version 2.15.0) with an expected value e-value of 1 × 10^−5^) via Diamond [18] (https://github.com/bbuchfink/diamond, version 2.0.13, accessed on 11 February 2026). KEGG functions corresponding to genes were obtained, and the abundance of functional categories was calculated by summing the gene abundances corresponding to KO, Pathway, EC, and Module. Regarding the identification of glucose stereoisomers (L- and D-glucose), it is important to note that standard GC-MS analysis without chiral columns cannot chemically resolve enantiomers. In this study, the chromatographic peak was identified as “Glucose” based on the retention index (RI) and mass spectral match against the standard library. However, considering that D-glucose is the exclusive biologically active form in Oryza sativa (rice) metabolism, and based on the database match (HMDB/KEGG), which primarily lists the D-form, the metabolite has been annotated as “D-Glucose” to reflect the expected biological stereochemistry. The “D-“ designation is thus assigned based on biological plausibility and database convention rather than direct chiral resolution by the instrument.

## 3. Results

### 3.1. Changes in Rice Fatty Acid Content

**Figure 1:**
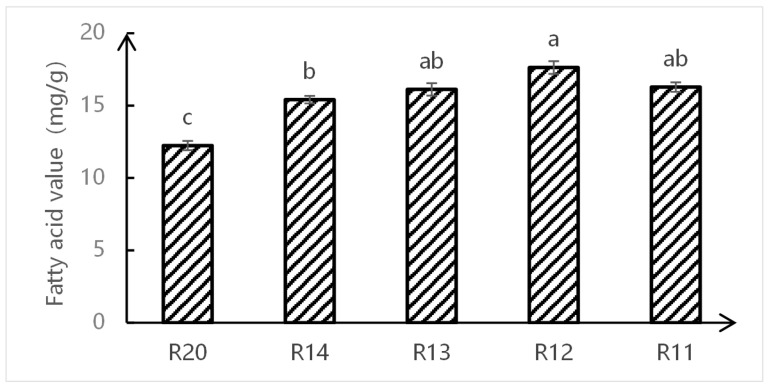
Changes in fatty acid values of japonica rice during storage. R20 (initial control, 0 months), R14 (4 months), R13 (8 months), R12 (12 months), and R11 (16 months); different lowercase letters above the bars indicate significant differences at *p* < 0.05 (one-way ANOVA followed by Tukey’s test).

Fatty acid value is a core physical and chemical indicator for measuring the deterioration degree of rice storage quality, and its dynamic change directly reflects the intensity of lipid hydrolysis and oxidation reactions [19]. The changes in fatty acid values of rice during different storage periods are shown in Figure 1. Statistical analysis revealed significant variations among the samples (*p* < 0.05). The fatty acid values of samples from R20 to R12 exhibited a significant upward trend, increasing from 12.24 mg/g (group c) to a peak of 17.63 mg/g (group a). Specifically, the value at R12 was significantly higher than that of the initial stage (R20). By the later stage of storage, the value for R11 slightly decreased to 16.28 mg/g; however, this difference compared to R12 and R13 was not statistically significant (sharing the same letter group), indicating a stabilization or slight fluctuation in lipid hydrolysis rates. The changes in fatty acid values of rice during different storage periods are shown in Figure 1. The fatty acid values of samples R20 to R12 exhibited an upward trend, increasing from 12.24 mg/g to 17.63 mg/g; by the later stage of storage, R11 slightly decreased to 16.28 mg/g. Rice is rich in lipids and prone to the degradation and release of fatty acids, leading to elevated fatty acid values, which can reflect the degree of quality deterioration. Field monitoring studies also indicate that the increase in fatty acid values during storage is strongly negatively correlated with quality, consistent with the present results. The changes in fatty acid values are influenced by storage environment, gas composition, moisture, and other factors. Studies on the quality of hybrid rice under different storage conditions show that fatty acid values initially increase and then stabilize in the later stage, which aligns with the results of this experiment. Therefore, the quality of samples R20, R14, R13, and R12 showed a downward trend, while R11 stabilized. Consequently, samples R20, R14, R13, and R12 were selected for subsequent non-targeted metabolomics and metagenomics analyses.

These findings indicate elevated activities of lipase and lipoxygenase during this phase, which accelerated the hydrolysis of triglycerides into free fatty acids (FFAs) [20]. In the late storage stage (R11), the FFA content slightly decreased to 16.28 mg/g. This decline is attributed to the further oxidative degradation of long-chain FFAs into small-molecule volatile aldehydes and ketones, as well as their participation in secondary metabolic reactions. Consequently, the mechanism of rice quality deterioration shifts from simple hydrolysis to complex oxidation and polymerization reactions [21]. Previous studies have highlighted that FFA accumulation not only compromises eating quality—manifested as reduced viscosity and the development of rancid off-flavors—but also serves as a critical marker for the loss of cell membrane integrity [22].

In our study, the rapid increase in FFAs observed from R20 to R12 closely coincided with peak periods of rice respiration intensity and endogenous enzyme activity. Conversely, the stabilization observed at R11 likely corresponds to substrate depletion or the inhibition of enzymatic activity. Based on the critical thresholds of quality deterioration revealed by FFA dynamics, four key sampling points representing distinct deterioration gradients were selected for subsequent integrated non-targeted metabolomics and metagenomics analysis: R20 (fresh), R14 (early-stage deterioration), R13 (mid-stage deterioration), and R12 (severe deterioration).

### 3.2. Analysis of Rice Metabolite Components

**Figure 2:**
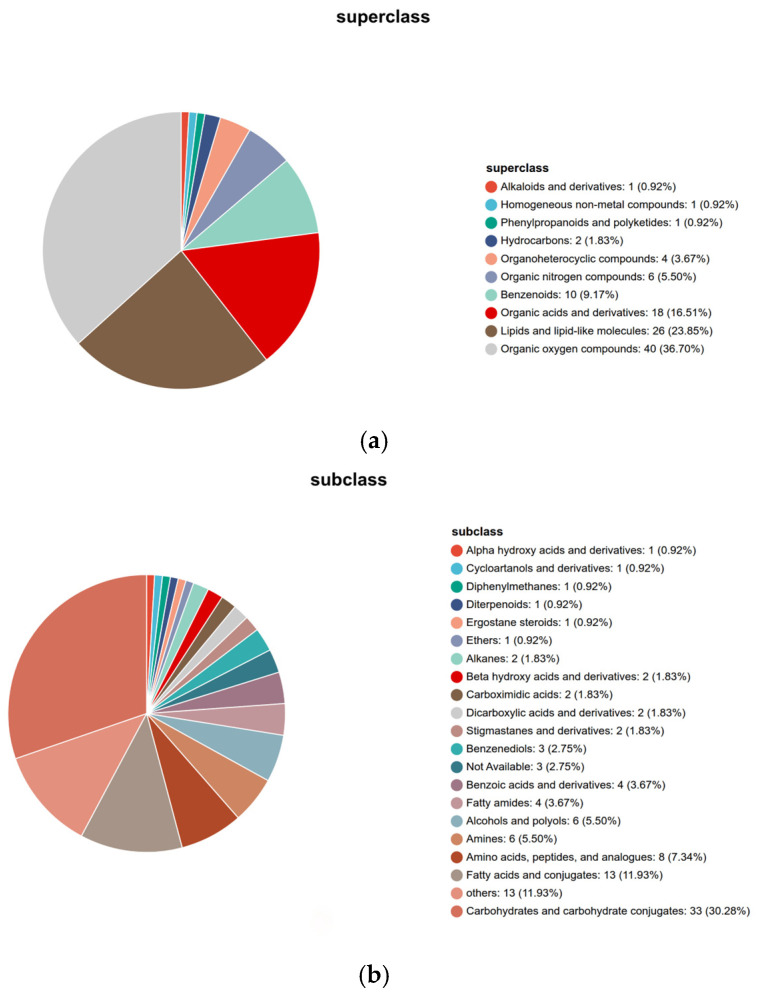
HMDB chemical taxonomy classification of all identified metabolites across all japonica rice storage samples (R20–R11). (**a**) Metabolite classification pie chart at the Superclass level. (**b**) Metabolite classification pie chart at the Subclass level. The charts illustrate the chemical diversity of metabolites detected under different storage durations.

A total of 264 cationic mass spectrometry peaks were detected and identified through comprehensive databases (Metlin, HMDB, etc.), yielding 263 highly credible metabolites (now provided in Supplementary Table S1). To characterize the chemical composition of the rice samples under various storage treatments, we annotated the detected features against the HMDB database. The Human Metabolome Database (HMDB), established in 2007 by The Metabolomics Innovation Centre (TMIC) in Canada, is considered the largest and most complete bio-specific metabolomics database in the world. By entering the HMDB ID number into the Search bar of the HMDB website, detailed information regarding the substance appears, including metabolite name, description, structure, molecular formula, molecular weight, and CAS number. Furthermore, each metabolite is assigned a specific classification within the database, which aids in further understanding the biological significance of key metabolites. Figure 2 presents the taxonomic distribution of the 264 metabolites identified across all experimental groups. Based on the HMDB hierarchical classification (Superclass, Class, Subclass) (Figure 2a), the Superclass level primarily includes: benzene ring compounds (10 species), organic acids and their derivatives (10 species), lipids and lipid-like molecules (24 species), oxygen-containing organic compounds (38 species), nitrogen-containing heterocyclic compounds (6 species), hydrocarbons (2 species), and alkaloids and their derivatives (1 species). Oxygen-containing organic compounds (38 species) and lipids and lipid-like molecules (24 species) collectively account for 23.6%, consistent with observations by Callier, M.D et al. [17] in geographically indicated rice, reflecting the core characteristics of the rice metabolome. Oxygen-containing organic compounds, predominantly sugars and alcohols, indicate that stored rice maintains basal metabolism to cope with stress. Lipids and lipid-like molecules, as core components of cell membranes, confirm Capuano, E et al. [18]’s conclusion that lipids influence anti-oxidation capacity by maintaining membrane structural stability, explaining the stable presence of these metabolites during storage. Organic acids and their derivatives (10 species) and benzene ring compounds (10 species) are functionally critical: organic acids participate in core pathways such as the tricarboxylic acid cycle, and their stable presence suggests that energy pathways remain partially active. Benzene ring compounds (mostly phenols) delay lipid oxidation by scavenging ROS, aligning with the phenolic protective effects observed by Steinhaus, M., and Schieberle, P. [22]. Nitrogen-containing heterocyclic compounds (6 types) and alkaloids with their derivatives (1 type) may participate in nitrogen metabolic balance, providing a foundation for protein stability during storage [23]. The diverse unsaturated fatty acids (e.g., linoleic acid and oleic acid) and their oxidative precursors identified in Table S1 serve as primary substrates for lipid peroxidation, indicating that lipid metabolic pathways underwent the most severe perturbation. Integrating recent multiomics insights, lipid degradation is regulated not only by endogenous enzymes but is also closely intertwined with the structure of microbial communities adhering to the grain surface [24]. Furthermore, the various organic acids detected, such as citric acid and succinic acid, are key intermediates of the tricarboxylic acid (TCA) cycle. Fluctuations in their levels reflect a metabolic shift in energy production during storage, transitioning from aerobic respiration toward anaerobic fermentation or hypoxic adaptation. Concurrently, the dynamic changes observed in aromatic compounds (e.g., vanillin and 4-hydroxybenzoic acid), which are downstream products of phenolic acid metabolism, suggest an activation of lignin biosynthesis pathways. This response likely functions to reinforce cell wall integrity as a defense mechanism against external stressors [25].

Subclass-level statistics (Figure 2b) reveal that carbohydrates (31 types) dominate, consistent with the metabolic characteristics underlying the formation of superior rice flavor profiles. These metabolites—including starch, oligosaccharides, and sugar alcohols—not only serve as primary energy sources but also exhibit composition-specific effects on rice gelatinization properties and taste scores. For instance, oligosaccharide content positively correlates with viscoelasticity, while sugar alcohols enhance textural refinement [26]. Fatty amides (3 types), as significant lipid derivatives, demonstrate higher stability than free fatty acids. They mitigate the formation of oxidative byproducts such as hexanal by inhibiting lipoxygenase activity, thereby delaying the “rancid flavor” [27]. Alcohols (6 types) and ethers (1 type) may participate in osmotic pressure regulation and antioxidant defense mechanisms, with their diversity reflecting the multi-pathway nature of metabolic regulation during storage. The persistent presence of fatty amides in this study suggests the existence of an intrinsic lipid oxidation defense mechanism within the rice grains.

**Figure 3:**
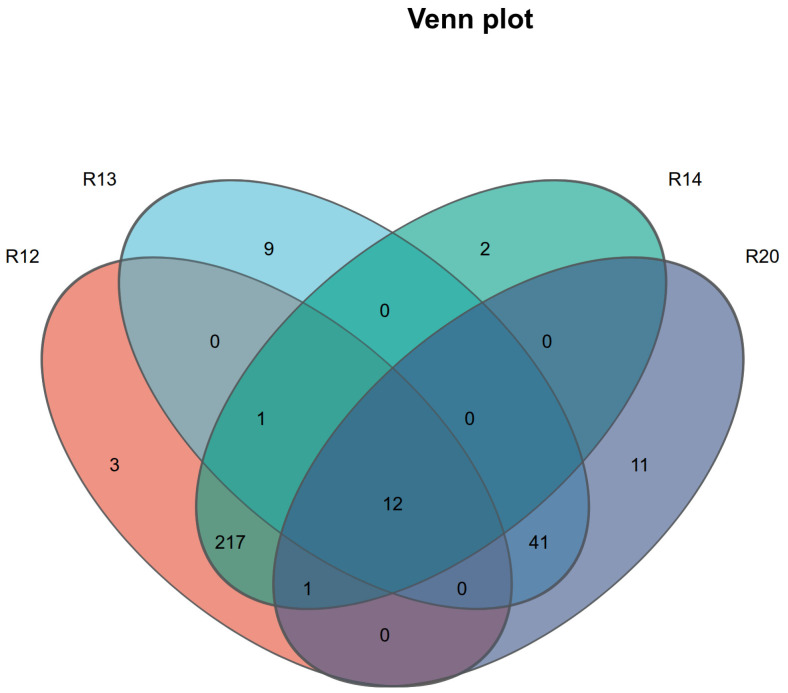
Venn diagram illustrating the overlap and unique numbers of metabolites detected in japonica rice samples at different storage durations. The codes represent specific storage time points: R20 (initial control, 0 months), R14 (4 months), R13 (8 months), and R12 (12 months).

The Venn diagram (Figure 3) statistically analyzes the number of shared and unique metabolites across different storage periods: R20 (initial control, 0 months), R14 (4 months), R13 (8 months), and R12 (12 months), with R12 containing 12 shared metabolites. R20 had 11 unique compounds, R14 had 2, R13 had 9, and R12 had 3. This variation pattern aligns with the metabolic dynamics during storage: In the initial stage (R20), fresh rice grains are rich in specific metabolites accumulated during the growth phase (e.g., antioxidant phenols and stress-resistant sugar alcohols), hence the abundance of unique metabolites; as storage progresses (R14), some specific metabolites undergo oxidative degradation or transformation, reducing their unique quantity to the minimum; in the middle and late stages (R13), rice grains initiate stress metabolism, generating degradation products or new defensive metabolites, leading to a rebound in unique metabolites; in the final stage (R12), metabolic activity slows down, retaining only a small number of stable unique metabolites, consistent with Langos, D. and Piironen, V’s conclusion [27] that metabolite types stabilize during the later storage period.

### 3.3. Analysis of Metabolite Composition Differences Among Rice Sample Groups

**Figure 4:**
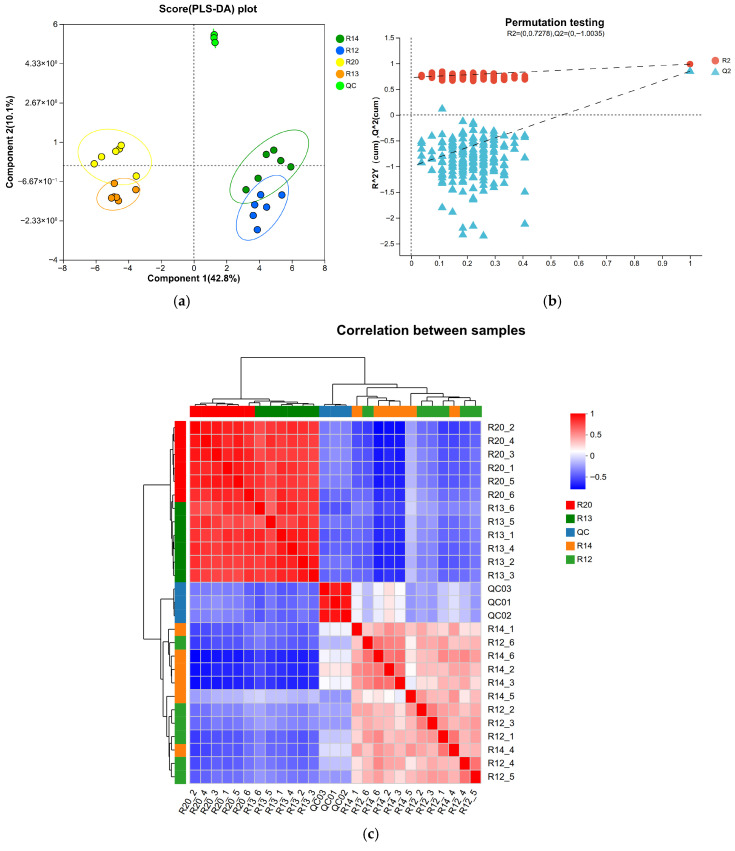
PLS-DA scores plots (**a**), PLS-DA permutation test (**b**), and heat-map and cluster analysis of metabolites (**c**).

Partial least squares discriminant analysis (PLS-DA) is a supervised multivariate statistical method commonly used for classification and discrimination problems. When the differences between sample groups are significant and the intra-group variation is small, unsupervised analysis methods can effectively distinguish between group characteristics. However, PLS-DA can further enhance the identification of inter-group differences by reinforcing the predefined classification objectives. The scatter plot obtained from PLS-DA serves as the core basis for intuitively evaluating the model’s classification performance, where more pronounced separation between sample groups indicates superior classification results [28]. Figure 4 includes the scatter plot (a), permutation test plot (b), and metabolic correlation heat map clustering analysis (c) obtained from PLS-DA. The horizontal and vertical coordinates of the scatter plot correspond to the first principal component (Component 1) and the second principal component (Component 2), respectively, with their numerical values representing the proportion of variance explained by each principal component in the original data.

As shown in Figure 4a, significant metabolic variations were observed between the quality control (QC) group and the four experimental rice groups (R20, R14, R13, and R12) at different storage stages. The metabolic profiles of rice samples exhibited clear separation in the score plots: the metabolite ion patterns of R20 and R14, R14 and R13, and R13 and R12 were distributed on opposite sides of the coordinate axes, achieving complete separation. This indicates that the metabolic profiles of rice at different storage stages show significant differences, and the impact of storage time on the composition of rice metabolites is statistically significant. The variance explained by the first and second principal components was 42.8% and 10.1%, respectively, cumulatively explaining 52.9% of the variance in the original data, demonstrating that the model effectively captures core metabolic differences between samples. To validate the reliability and predictive power of the PLS-DA model, a permutation test (default number of permutations) was performed, as shown in Figure 4b: the intercept of the Q^2^ regression line with the *y*-axis was −0.8972, and the model evaluation parameter R^2^ = 0.7221, with a Q^2^ value close to 1, indicating excellent model fit and strong predictive ability without overfitting. This provides reliable support for the subsequent screening and identification of inter-group differential metabolites [29]. The PLS-DA model constructed in this study demonstrated excellent classification performance, with complete separation of rice samples across groups, confirming that storage time is a key driver of rice metabolome evolution. The first principal component accounts for 42.8% of the high variance explained, indicating persistent changes in a core set of metabolites during storage. These metabolites may serve as key targets regulating the evolution of rice quality. The permutation test results (Q^2^ intercept = −0.8972) meet the reliability criteria for metabolomics models, significantly below the 0.05 threshold, further confirming that the model is free from random interference. The captured metabolic differences authentically reflect the essential characteristics of different storage stages [30], consistent with the validation criteria for the PLS-DA model established by Liu et al. [31] in rice metabolomics research.

The heat map of metabolite correlations between samples, combined with cluster analysis, can quantitatively characterize the variation features of metabolite composition and abundance across different samples. In this study, after preprocessing the data with Pareto transformation, the Pearson correlation algorithm was used to calculate the inter-sample correlation degree, followed by cluster analysis at the Complete linkage level. A correlation coefficient closer to 1 indicates higher similarity in metabolite composition and abundance between samples. As shown in Figure 4c, rice samples R20 and R13 exhibit a strong positive correlation with a correlation coefficient of 0.62, suggesting high similarity in metabolite composition between these two storage-stage rice samples. Although samples R14 and R12 show a positive correlation, their correlation coefficient is only 0.16, indicating weak correlation and significant differentiation in their metabolic profiles. Additionally, QC samples demonstrate clustered characteristics in the heat map, further validating the stability of the experimental procedures and data reproducibility, thereby ensuring the reliability of inter-group difference analysis results [32]. The inter-sample correlation analysis results complementarily validate the clustering patterns observed in previous PCA and PLS-DA analyses: The high correlation between R20 and R13 (r = 0.62) suggests that rice samples from the early storage stage (R20) and mid-to-late stages (R13) may retain some conserved metabolic pathways. This continuity in metabolic characteristics is likely closely related to the relative stability of rice quality. Existing studies have demonstrated that during grain storage, the conservation of core metabolic pathways is often accompanied by the sustained accumulation of antioxidant metabolites, which effectively delay lipid oxidation and carbohydrate degradation, thereby maintaining quality stability. The weak correlation between R14 and R12 (r = 0.16) indicates a significant shift in the metabolic regulation pattern of rice during these two stages, which may be associated with accelerated quality deterioration from the mid to late storage phase. This is hypothesized to involve core processes such as the accumulation of lipid peroxidation products and the degradation of key nutritional metabolites [33].

### 3.4. KEGG Differential Metabolite Analysis

**Figure 5:**
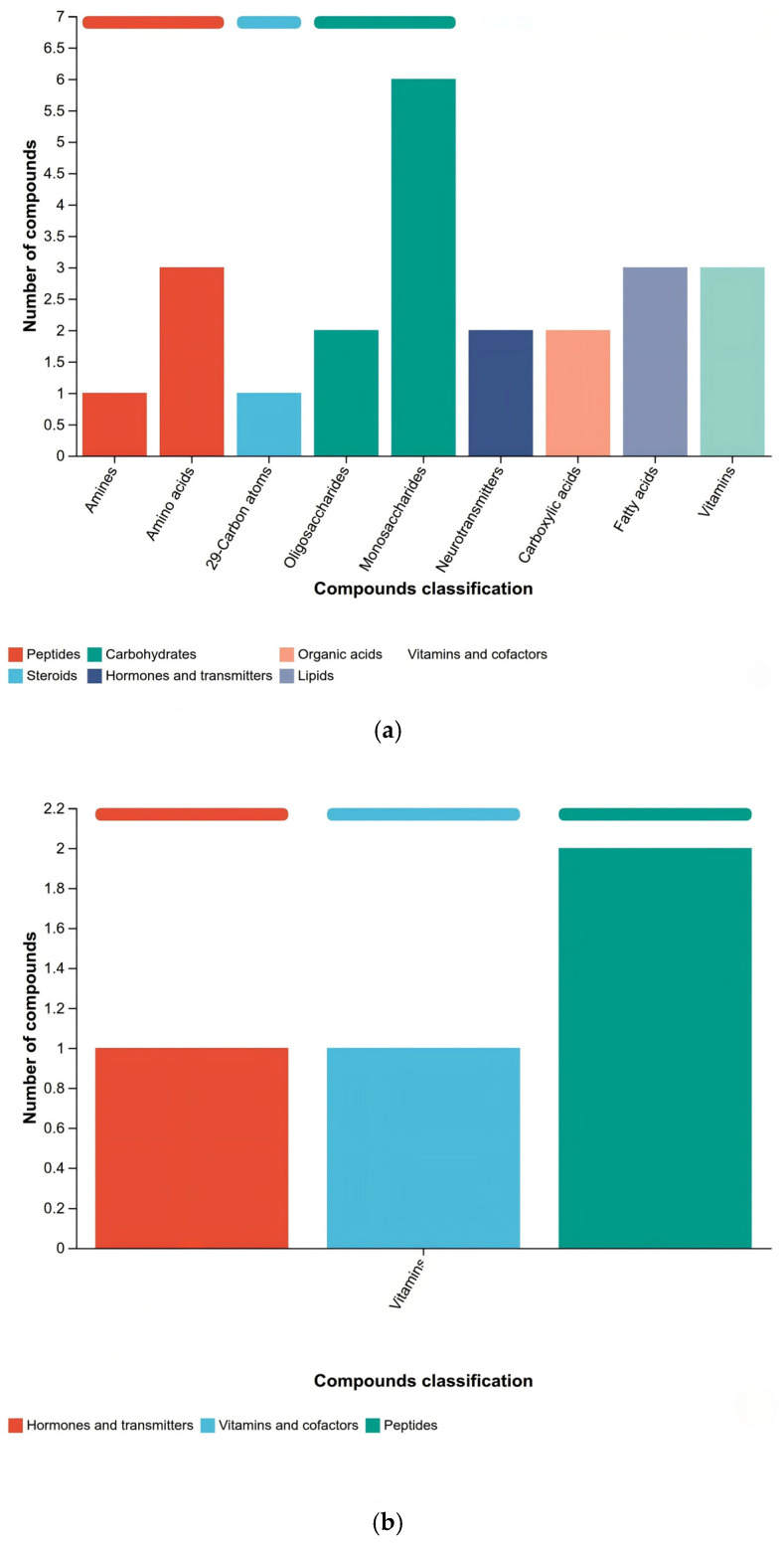

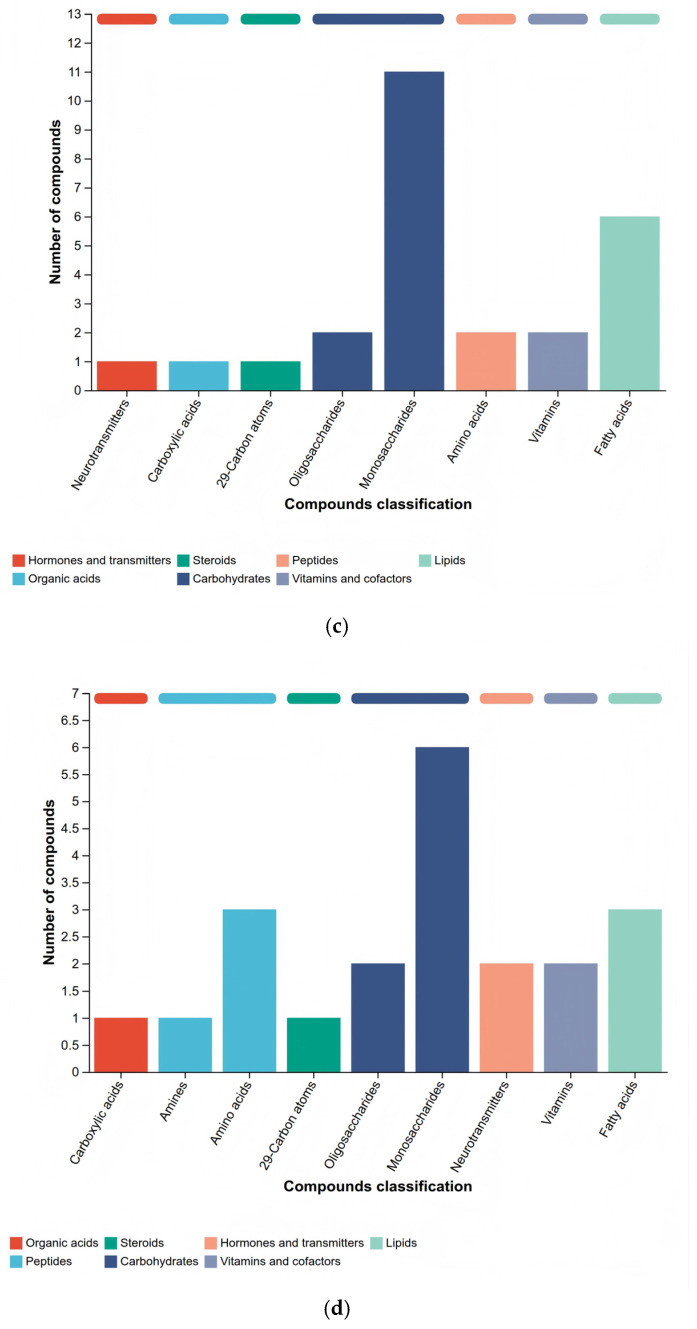
KEGG compound classification between two groups: (**a**) R12-R13; (**b**) R12-R14; (**c**) R12-R20; and (**d**) R13-R14.

KEGG compound classification categorizes metabolites based on their structural characteristics and physicochemical properties into classes such as carbohydrates, amino acids, organic acids, and lipids, with further classification according to biological functional hierarchy. Core categories include biofunction-related compounds, bioactive peptides, endocrine disruptors, pesticides, plant secondary metabolites, and lipids [28]. This study compared identified metabolites with the KEGG compound database to obtain an overview of their functional classifications and statistical mapping (Figure 5). Based on the differential analysis conclusions from Section 3.3 and Section 3.4, this study focused on sample groups with significant differences in metabolite composition and abundance, conducting an in-depth analysis of their KEGG functional classification differences. The subplots in Figure 5a–d correspond to the functional classification statistics of metabolites from four comparative groups: R12/R13, R12/R14, R12/R20, and R13/R14. The *x*-axis represents the secondary functional classifications of KEGG compounds, while the *y*-axis shows the number of metabolites annotated to each category. According to biological functions, the differentially expressed metabolites were primarily classified into nine major categories: organic acids, lipids, carbohydrates, nucleic acids, peptides, vitamins and coenzymes, steroids, hormones and signaling mediators, and antibiotics.

The KEGG differential metabolite profile analysis revealed that among the three R12/R13, R12/R20, and R13-R14 comparisons, the predominant differential metabolites were monosaccharides within the carbohydrate class. Significant variations were also observed in peptides, steroids, organic acids, hormones and signaling molecules, lipids, vitamins, and coenzyme factors. In contrast, the R12-R14 group exhibited a distinct pattern, with amino acids being the primary metabolites in peptides, while only minor amounts were present in hormones, signaling molecules, vitamins, and coenzymes. Notably, compared to the initial storage stage (R20), the R12 samples stored for 12 months showed a marked enrichment of monosaccharides in carbohydrates and fatty acids in lipids. This distribution pattern aligns closely with the peak fatty acid levels observed in R12 samples as described in Section 3.1, confirming the correlation between functional classification of metabolites and rice quality indicators.

KEGG functional classification analysis revealed the core metabolic differences in rice grains during different storage periods at the metabolic pathway level. The concentrated distribution of differential metabolites in carbohydrate, lipid, and peptide categories reflects the dynamic transformation of core metabolic pathways during grain storage, which is consistent with the observation by Wang, Chen et al. [19] in cereal storage metabolomics research that “core nutrient metabolic pathways dominate quality evolution.” Carbohydrate monosaccharides, as the primary category of differential metabolites, exhibit changes closely related to energy metabolism and quality deterioration during storage. During storage, polysaccharides such as starch gradually degrade into monosaccharides, which not only provide energy for the grain’s own respiration but also accumulate monosaccharides that may undergo Maillard reactions to generate off-flavor compounds, affecting edible quality [34]. The enrichment of carbohydrate monosaccharides in R12 samples suggests intensified starch degradation during the late storage phase, aligning with the conclusion that starch gelatinization characteristics deteriorate during storage in high-quality rice. The differential distribution of fatty acids in lipids is of significant importance. The peak fatty acid content in R12 samples corresponds to the enrichment of fatty acid metabolites in KEGG classification, while the increase in fatty acid content directly reflects lipid oxidation during storage—unsaturated fatty acid oxidation and decomposition produce off-flavor compounds such as hexanal and nonanal, which are the core factors leading to flavor deterioration in rice. This finding is consistent with the conclusion by Shi, S. et al. that “fatty acid oxidation is a key driver of quality decline in stored grains,” as confirmed by lipidomics. The differential distribution of amino acids among peptides reflects the dynamic changes in protein metabolism during rice storage. During storage, proteins gradually degrade into amino acids, which may lead to a decrease in the viscoelasticity of rice and a deterioration in its texture. On the other hand, the loss of certain essential amino acids may reduce the nutritional value of rice [35]. The differential metabolites in groups R12-R14 were predominantly amino acids, suggesting significant differences in protein degradation rates during these two storage stages, which may be related to variations in microbial activity or enzyme activity in the storage environment [36]. The diversity of functional categories of differential metabolites across different comparison groups indicates the complexity of metabolic regulation during rice storage—beyond the core metabolism of carbohydrates, lipids, and proteins, differences in vitamins and coenzymes, hormones, and signaling mediators also reflect dynamic adjustments in stress metabolism and signal regulation. For example, differences in vitamins and coenzymes may be associated with the activation of the rice’s antioxidant defense system, and their content changes can indirectly reflect the intensity of oxidative stress during storage [37].

### 3.5. Metabolic Pathway Analysis of Differential Metabolites

**Figure 6:**
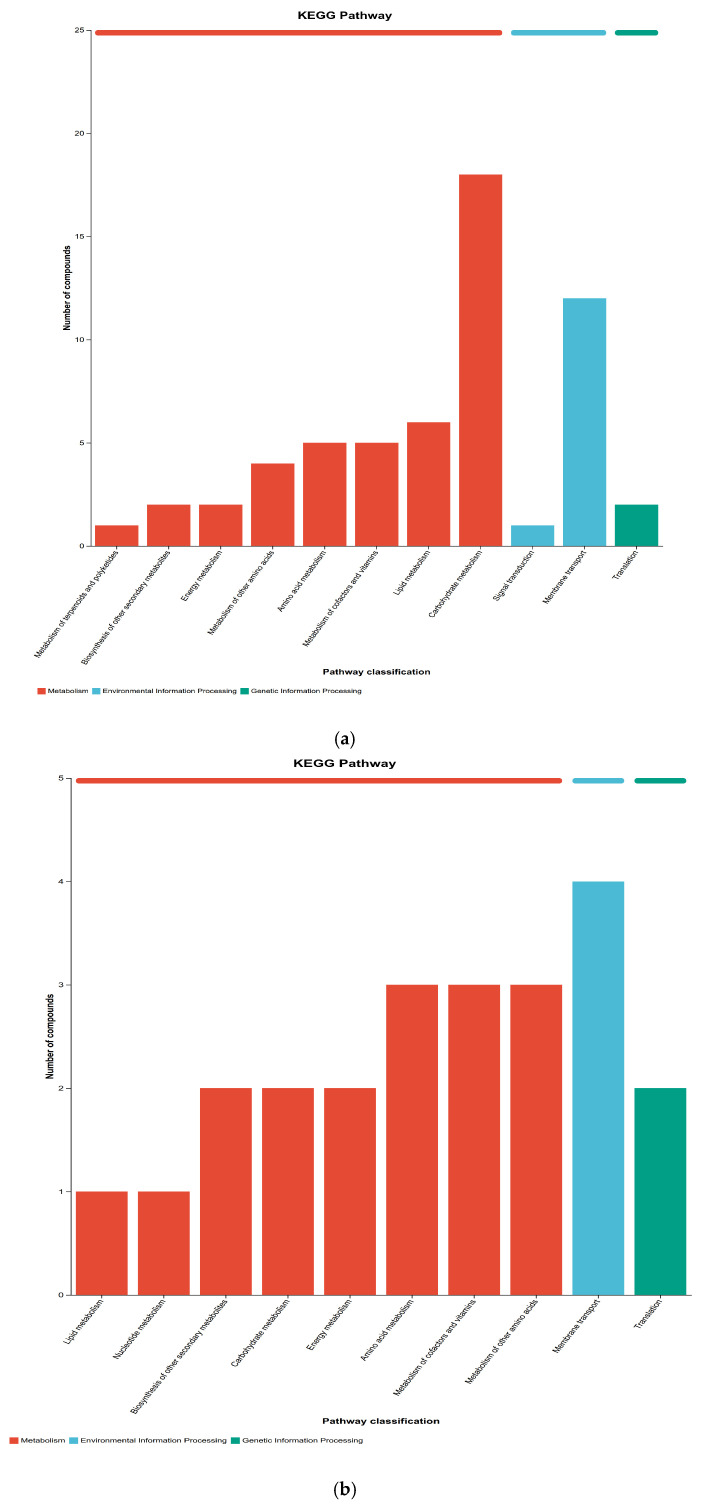

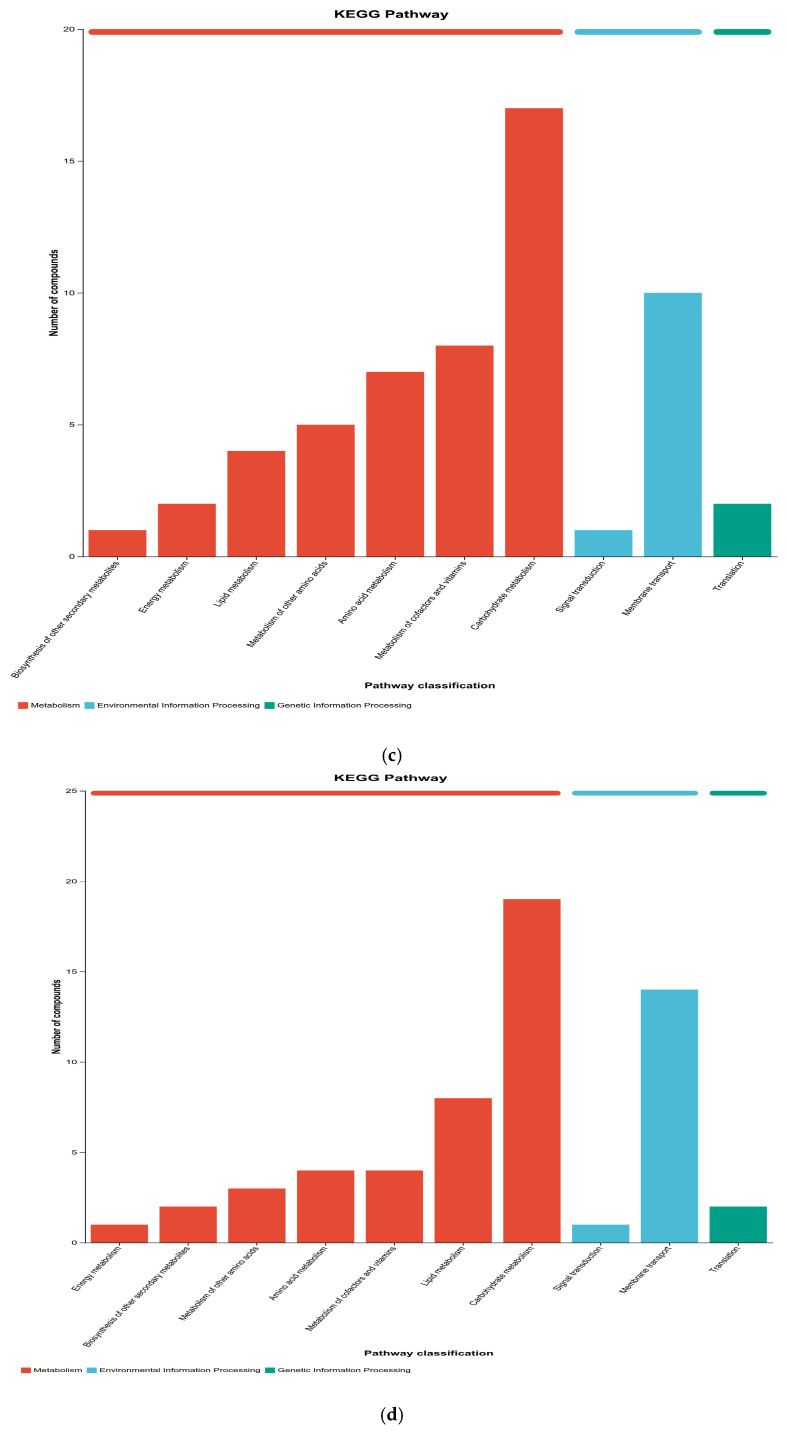
KEGG pathways, Level 2, between two groups: (**a**) R13-R14; (**b**) R12-R14; (**c**) R12-R13; and (**d**) R12-R20.

The KEGG PATHWAY database contains a manually curated collection of metabolic pathways, systematically describing molecular interactions, physiological and biochemical reactions, and regulatory relationships among gene products. Based on the alignment results between metabolites and KEGG compounds, this study identified core metabolic pathways involved in differential metabolites and evaluated their regulatory roles in the overall metabolic processes of rice. In Figure 6, the *x*-axis represents the secondary classifications of KEGG metabolic pathways, while the *y*-axis indicates the number of compounds annotated to the corresponding pathways. In Figure 6, the *x*-axis represents the secondary classifications of KEGG metabolic pathways, while the *y*-axis indicates the number of compounds annotated to the corresponding pathways. Higher enrichment levels of compounds suggest more critical roles of these pathways in metabolic regulation during the storage period of rice. KEGG metabolic pathways are categorized into seven major classes: metabolism, genetic information processing, environmental information processing, cellular processes, organismal systems, human diseases, and drug development, with different classes distinguished by color-coded annotations.

The differential metabolites were annotated to KEGG secondary metabolic pathways to identify inter-group metabolic pathway enrichment features. Results showed (Figure 6a) that in the comparison between R13 and R14 groups, the pathways with higher enrichment of differential metabolites were annotated to 12 and 18 compounds, respectively, reflecting significant differentiation in rice metabolic pathways between these two storage stages. In the comparison between the R20 (freshly harvested rice) and R12 (stored rice for 12 months) groups (Figure 6d), the pathways with the highest enrichment of differential metabolites were transmembrane transport and carbohydrate metabolism pathways, with 19 and 14 compounds, respectively, which became the core metabolic pathways of the two sample groups. As shown in Table 1, the differential metabolic pathways between the R20 and R12 groups were primarily concentrated in five major categories: amino acid metabolism, flavonoid synthesis, short-chain fatty acid metabolism, energy metabolism, and lipid metabolism. Eleven key differential metabolites were identified, including 4-dihydroxymandelic acid (3,4-Dihydroxymandelic Acid), 4-coumaric acid (4-Coumaric acid), 9-octadecenoic acid (9-Octadecenoic acid), citric acid (Citric Acid), D-glucose (Free D-Glucose), L-asparagine (L-Asparagine), L-glutamic acid (L-Glutamic Acid), myristic acid (Myristic acid), palmitic acid (Palmitic Acid), ribose (Ribose), and stearic acid (Stearic Acid). Compared to the initial sample R20, the significantly up-regulated metabolites in R12 samples were 4-dihydroxymandelic acid, citric acid, D-glucose, L-asparagine, L-glutamic acid, and ribose, indicating a significant enrichment trend of these metabolites in the late storage phase of rice grains. The significantly down-regulated metabolites were 4-coumaric acid, 9-octadecenoic acid, D-glucose (Free D-Glucose), myristic acid, palmitic acid, and stearic acid. The fluctuations in the content of these metabolites not only directly affect the edible quality of rice grains but may also indirectly lead to changes in the fungal community structure of rice grains [38]. The high enrichment of transmembrane transport and carbohydrate metabolism pathways in the R20 and R12 groups suggests significant adjustments in material transport efficiency and energy metabolism patterns during grain storage. The activation of transmembrane transport pathways may be related to the material exchange demands of rice in response to storage environmental stresses (e.g., water changes, microbial infections), while the differences in carbohydrate metabolism pathways reflect the degradation and transformation of energy-storing substances such as starch [39]. This aligns with the “carbohydrate metabolism-dominated quality evolution” observed by Coton, M. et al. in rice storage metabolomics studies. The up-regulation of amino acids such as L-asparagine and L-glutamate may stem from the hydrolytic degradation of proteins during storage—proteins gradually break down into amino acids to maintain basal metabolic activities. However, excessive accumulation of these amino acids can lead to reduced stickiness and elasticity of rice, resulting in poorer texture, which is consistent with the conclusion of Johnson, T.S. et al. [40] that “protein degradation during rice storage is negatively correlated with taste quality.” Additionally, as nitrogen sources, changes in amino acid content may influence the growth and reproduction of microorganisms such as fungi, thereby altering the fungal community structure of rice. This association provides a new perspective for understanding the mechanisms of mold formation during storage [41]. The down-regulation of fatty acids such as palmitic acid, stearic acid, and 9-icosapentaenoic acid may be related to lipid oxidation during storage—unsaturated fatty acid oxidation produces off-flavor compounds such as hexanal and nonanal, which are key factors contributing to the deterioration of rice flavor. The decline in the content of these fatty acids, along with the peak fatty acid values observed in the R12 samples mentioned earlier, reflects the dynamic equilibrium of lipid metabolism: fatty acids accumulate during initial storage due to lipid degradation (increased fatty acid values), while they decrease later due to continuous oxidative breakdown. This explanation aligns with the results of the lipidomics study by Liu et al. As a precursor to phenolic antioxidants, the reduction in 4-coumaric acid content leads to a decline in the rice’s ability to eliminate reactive oxygen species (ROS), accelerating the oxidative deterioration of lipids, carbohydrates, and other substances. This finding is consistent with the conclusion by Lee, S et al. that “phenolic substance content is positively correlated with grain storage stability.” Additionally, citric acid, a key intermediate in the tricarboxylic acid cycle (TCA cycle), shows up-regulated expression, suggesting enhanced energy metabolism in rice during the late storage phase, which may represent a stress response to environmental stressors [42]. The up-regulation of carbohydrates such as D-glucose (Free D-Glucose) and ribose further confirms the intensification of starch degradation metabolism—starch gradually breaks down into monosaccharides to provide energy for metabolic activities, but excessive accumulation can lead to the formation of undesirable flavor compounds like furans and pyrazines through the Maillard reaction, while also resulting in abnormal sweetness and poor gelatinization characteristics in rice.

### 3.6. Analysis of Differences in Microbial Community Structure Composition of Japonica Rice at Different Storage Periods

To elucidate the community structure characteristics of dominant microorganisms (relative abundance > 1%) in rice samples during different storage periods, this study employed metagenomic sequencing technology to obtain microbial community data. Statistical analysis methods were used to conduct differential analysis among diversity indices, and principal component analysis (PCoA) was employed to characterize overall community differences. Additionally, fungal community composition was annotated at the taxonomic levels of phylum, genus, and species. Non-parametric tests were used to screen differential species, and linear discriminant analysis effect size (LEfSe) was applied to identify inter-group potential biomarkers, systematically analyzing the evolutionary patterns of fungal communities in stored rice.

**Figure 7:**
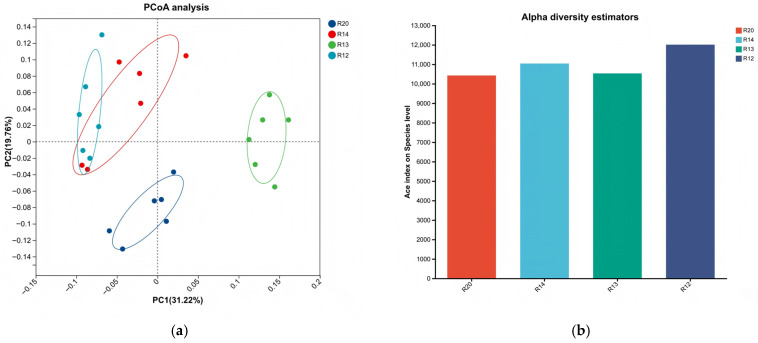

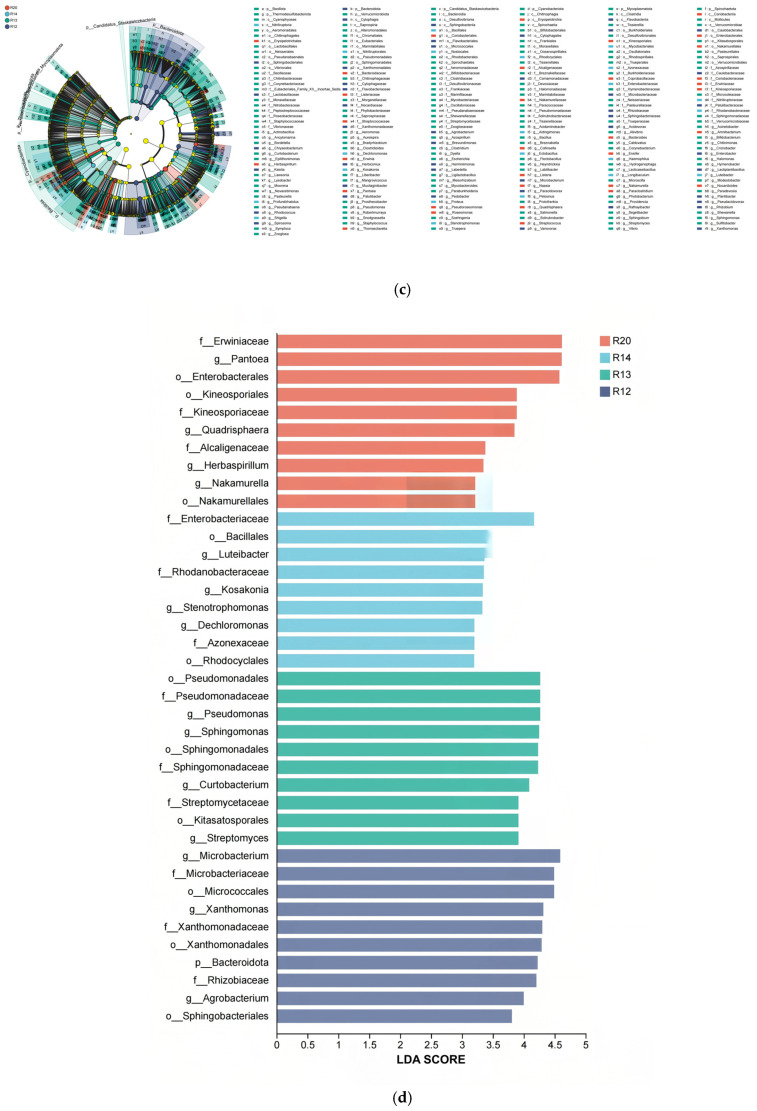
Comparison of fungal communities in japonica rice stored at different periods: (**a**) α-diversity analysis; (**b**) principal component analysis (PCoA) score plot; (**c**) LEfSe analysis; and (**d**) LDA score plot.

**Figure 8:**
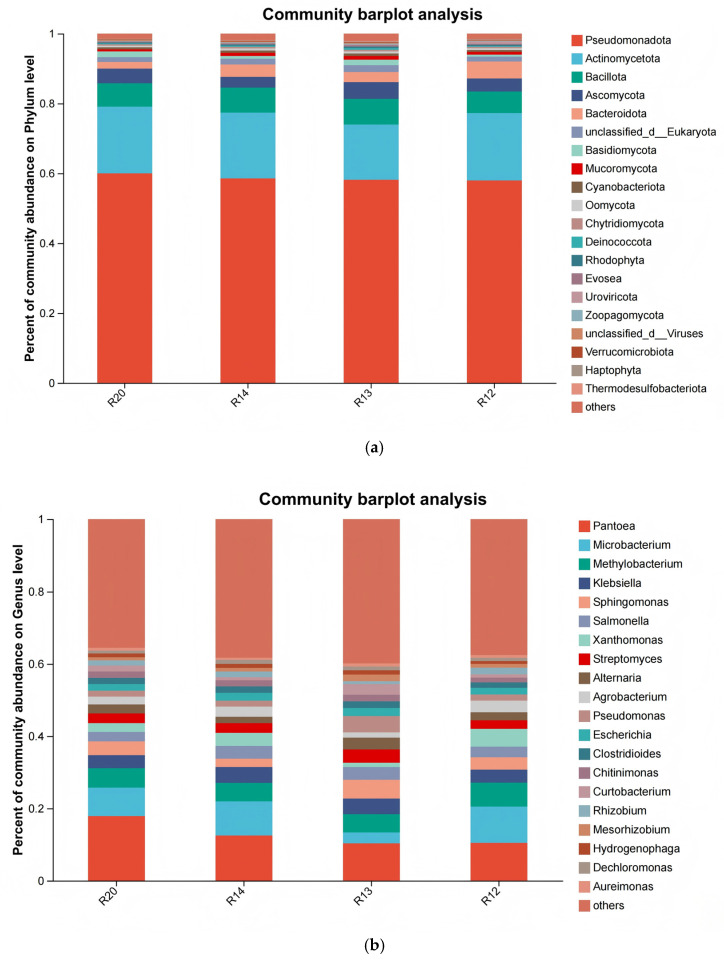

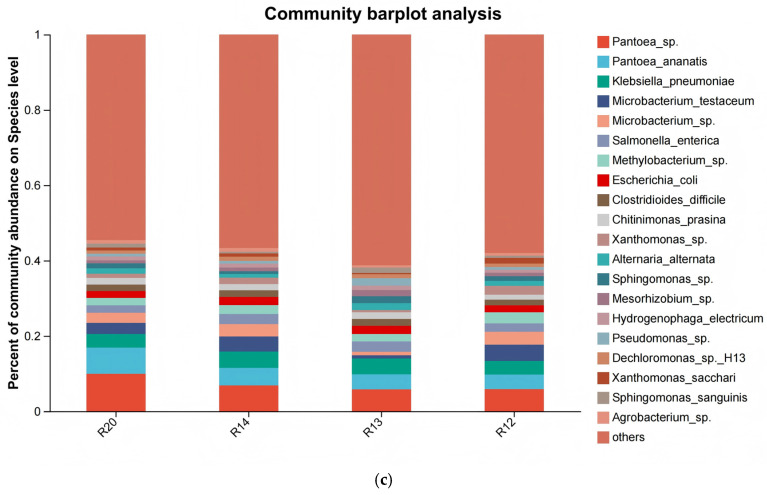
The microbial community composition of japonica rice samples at the phylum (**a**), genus (**b**), and species (**c**) levels.

The PCoA analysis (Figure 7a) revealed significant separation of fungal community compositions among the four sample groups, with R20 showing the most pronounced distinction from other stages, indicating a unique early community structure. The α-diversity analysis (Figure 7b) demonstrated that R12 exhibited the highest ACE index and the richest fungal diversity, consistent with the pattern of fungal proliferation and increased species richness observed with prolonged storage duration [43]. The high fungal diversity in R12 during the later storage period may be attributed to favorable conditions created by changes in rice moisture and nutrient degradation (e.g., starch hydrolysis providing sufficient carbon sources for monosaccharide formation) [44]. The phylum-level community composition (Figure 8a) revealed the dominant phyla (in descending abundance) as Pseudomonadota (60.2%), Actinomycetota, Bacillota, Ascomycota, and Bacteroidota. Pseudomonadota was the absolutely dominant phylum. Compared to R20, R12 showed significantly increased abundance of Actinomycetota and Bacteroidota, reflecting their proliferative dominance during the later stage [45]. The absolute dominance of Pseudomonadota aligns with the widespread distribution of Gram-negative bacteria in grain storage environments. Some species of the *Pseudomonas* genus possess extracellular enzyme activity, which can degrade rice proteins, lipids, and other nutrients, potentially accelerating quality deterioration [46]. At the genus level (Figure 8b), *Pantoea* was the dominant genus with a relative abundance of 38.19%, while Aeromonas exhibited extremely low abundance. The species-level analysis (Figure 8c) revealed that *Pantoea* sp., a dominant species (36.17%), was identified as a core potential biomarker of fungal communities during rice storage, with its abundance changes serving as a key indicator for characterizing the storage phase [47]. Some species of *Pantoea* are endophytic fungi, while others may become pathogenic under storage conditions. The sustained increase in their abundance may be associated with elevated mold risk [48]. Studies indicate that metabolites produced by *Pantoea* sp. can influence grain flavor, and their biomarker properties provide potential targets for developing rapid early-warning technologies for storage quality [49].

Non-parametric tests were employed to screen for differences in species abundance between groups: Kruskal–Wallis sum-rank test was used for multi-group comparisons, and Wilcoxon rank-sum test for two-group comparisons. The consistency of differential species within subgroups was further validated by the Wilcoxon rank-sum test, combined with LEfSe analysis for potential biomarker identification (Figure 7c,d). A total of 38 clearly annotated differential potential biomarkers (excluding unannotated species) were identified, with LDA scores ranging from 0 to 4.61. Among these, 19 high-influence potential biomarkers with LDA scores > 4 played a critical role in distinguishing fungal communities at different storage stages [50]. *Pantoea* sp. exhibited a Linear Discriminant Analysis (LDA) score > 4.0, confirming its status as a key biomarker distinguishing fresh samples (R20) from those in later storage stages. The sustained increase in *Pantoea* abundance signifies not only the activation of endogenous microbial communities but also suggests a critical link to the development of “aging off-flavors” in rice. Specifically, volatile organic compounds (VOCs) and organic acids generated through *Pantoea* metabolism may directly contribute to the accumulation of undesirable sensory attributes [51]. Furthermore, while certain *Ascomycota fungi*, such as low-abundance species of *Aspergillus* and *Penicillium*, were specifically enriched at the R12 stage without becoming dominant taxa, their potential risk cannot be overlooked. The possible activation of their mycotoxin biosynthetic gene clusters warrants close monitoring during late-stage storage to ensure food safety [52]. The accumulation of amino acid metabolites during late storage may promote the proliferation of Bacteroides, while lipid oxidation products provide nutrients for Actinomycetes [53]. This “metabolite–microorganism” interaction collectively drives changes in rice storage quality [54].

### 3.7. Multiomics Association Analysis

The integrated analysis of multiomics can compensate for issues caused by data gaps and noise interference in single-omics data. Cross-validation among multiomics datasets reduces false positives from single-omics analysis, facilitating in-depth research on phenotypes and regulatory mechanisms in organisms [55]. Species/function distribution network analysis can demonstrate the distribution patterns between samples and species/function. Correlation analysis of species and functional abundance across different samples was performed to reveal co-occurrence relationships within environmental samples, highlighting similarities and differences among them. The resulting ecological networks were constructed and analyzed using Python-based tools, following established methodologies for microbial interaction inference. Species and KEGG functional contribution analysis is based on the correspondence between species and functions in samples, conducting correlation analysis between the relative abundance of species and functions to identify the functional contribution of specific species and the contribution of specific functions to species, thereby determining the functional bacterial genera that dominate KEGG metabolic pathways.

#### 3.7.1. Functional Distribution Network Analysis of Rice Fungal Species

**Figure 9:**
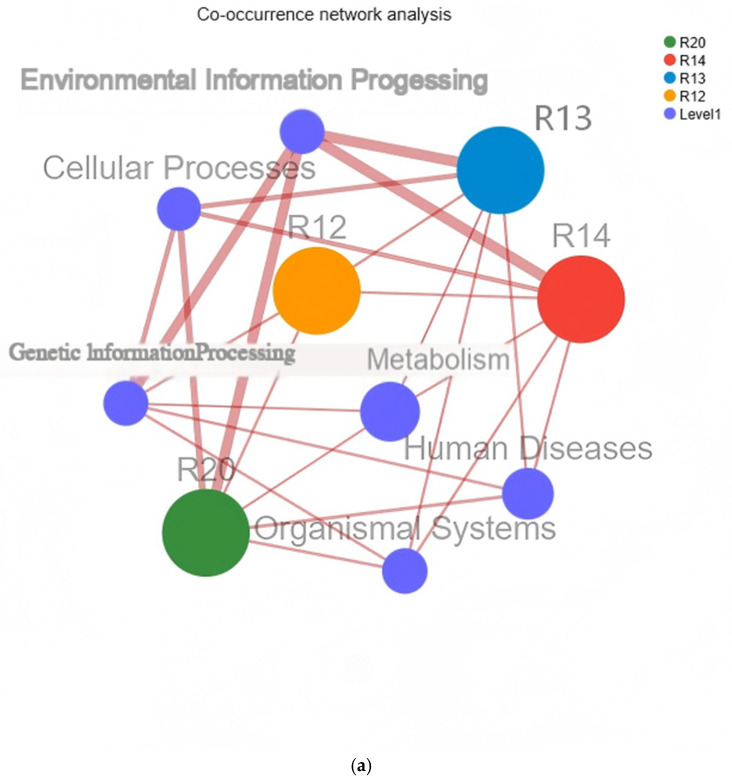

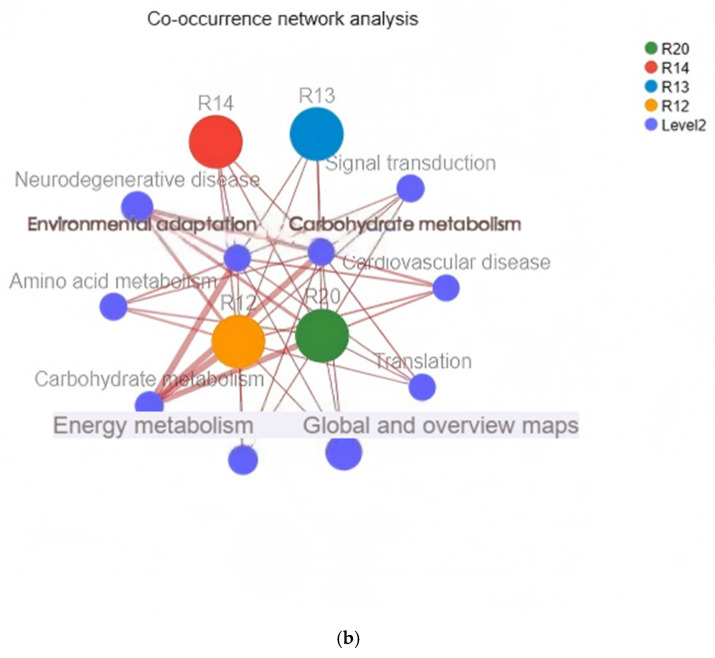
KEGG co-occurrence network analysis on different levels: (**a**) Level 1 and (**b**) Level 2.

This analysis generates clusters based on shared species/function among samples, meaning the more shared species/function, the closer the relationships between samples. By calculating topological attributes such as degree distribution, network diameter, average shortest path, node connectivity (degree), closeness centrality, and betweenness centrality, it obtains relevant information about species/function and within-group or inter-group relationships [56]. The collinearity network illustrates the coexistence relationships between species and samples, facilitating the understanding of dominant species distribution across different samples. As shown in Figure 9a, at the KEGG Level 1 functional level, significant differences were observed in the associations between samples and functional modules such as Genetic Information Processing, Metabolism, and Human Diseases: R20 showed strong correlations with the Genetic Information Processing module, while R14 and R12 exhibited stronger associations with the Metabolism and Human Diseases modules. At the Level 2 functional level in Figure 9b, R20 was closely associated with Signal Transduction and Carbohydrate Metabolism modules, whereas R12 demonstrated distinct association patterns with Neurodegenerative Diseases and Cardiovascular Diseases modules, reflecting the differential distribution of fungal functions in rice during different storage periods [57]. The analysis of topological properties in the functional distribution network of species revealed the dynamic restructuring of fungal functions during storage in rice: in the early storage stage, the strong association between R20 and functional modules such as genetic information processing and signal transduction may be related to the low activity and low metabolic state of fungi in fresh rice, with their functions concentrated on maintaining basic life activities. In contrast, the strong association between R12 and metabolic and disease-related modules in the later storage stage suggests that fungi activated more metabolic pathways during proliferation, and some metabolites may adversely affect rice quality [58].

#### 3.7.2. Key Functional Bacterial Genus in KEGG Metabolic Pathways

**Figure 10:**
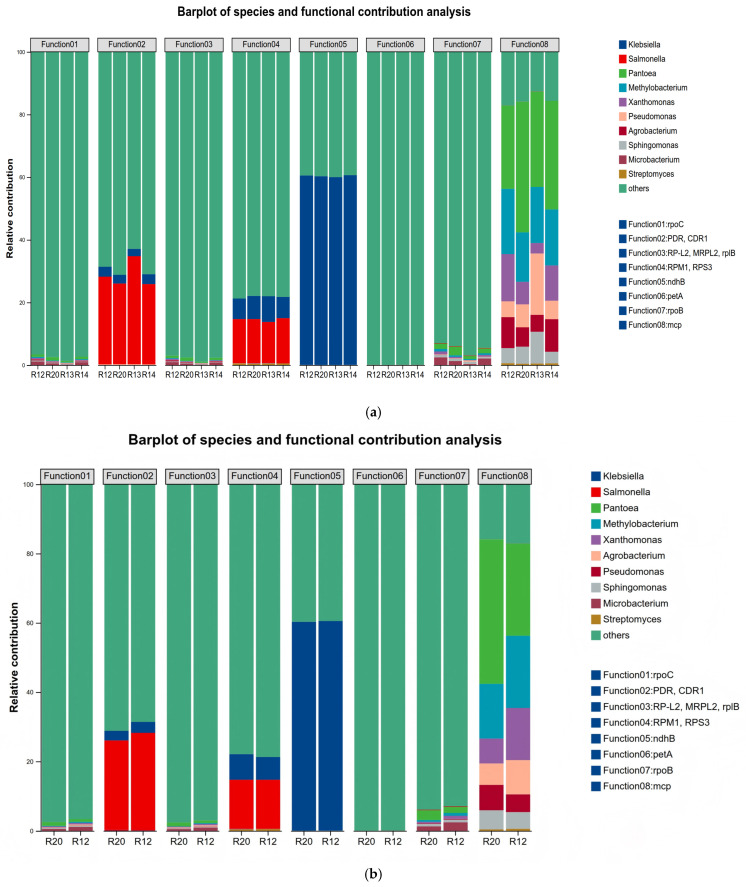
Barplot of species and functional contribution analysis of (**a**) all groups and of (**b**) R12-R20.

KEGG functional contribution analysis was conducted based on the abundance data of fungal genera and enriched key metabolites in rice samples, using Python (Version 2.7.0) for association analysis. This study screened the top ten most abundant genera and constructed association bar charts with the top eight KEGG functional pathways to identify the core fungal genera dominating key pathway functions during storage (Figure 10). The eight enriched key KEGG pathways were rpoC, PDR/CDR1, RP-L2/MRPL2/rplB, RPM1/RPS3, ndhB, petA, rpoB, and mcp. Functional contribution analysis revealed that *Klebsiella* was the sole dominant genus for the ndhB pathway function; *Salmonella* was the core functional genus for the PDR/CDR1 pathway; the RPM1/RPS3 pathway was co-dominant by *Klebsiella* and *Salmonella*; and the mcp pathway function was achieved through the synergistic action of *Pantoea*, *Methylobacterium*, *Xanthomonas*, *Pseudomonas*, *Agrobacterium*, and *Sphingomonas* [59]. The *Salmonella*-dominated PDR/CDR1 pathway is associated with drug resistance, suggesting that late-storage fungi may activate resistance pathways to adapt to environmental stress, thereby increasing control difficulty. The RPM1/RPS3 pathway, co-dominated by two fungal genera, involves plant defense responses. Its abnormal activation may disrupt the rice’s own defense system, further exacerbating quality loss. The MCP pathway depends on multi-genera synergy, which is related to chemotaxis. Multi-genera synergy may enhance fungi’s utilization efficiency of internal rice nutrients, accelerating spoilage during storage. The differential functional contributions of fungal genera at different storage stages reflect the phased characteristics of fungal community functions. The most significant functional differences between R20 and R12 align with the previously reported microbial community diversity and metabolic pathway evolution, confirming the chain-like association of “community structure–metabolic function–quality changes” [60]. The low contribution of Panagenus during early storage coincides with the stability of rice metabolites, while its enhanced synergistic contribution to the mcp pathway in late storage interacts with the accumulation of monosaccharides, amino acids, and other metabolites. The ndhB pathway, dominated by *Klebsiella*, is related to energy metabolism. Its activation may accelerate carbohydrate degradation in rice, leading to quality deterioration [61].

#### 3.7.3. Correlation Analysis Between Dominant Bacterial Communities and Major Volatile Compounds

**Figure 11:**
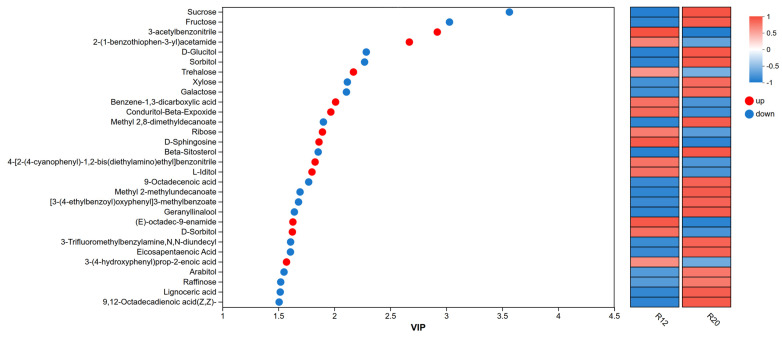
Variable importance in projection (VIP) scores of japonica rice samples R12 and R20. Note: The left panel displays a bubble plot of metabolites with VIP values, where the *y*-axis represents metabolites, and the *x*-axis shows VIP values. Metabolites are arranged from top to bottom according to their VIP values. The right panel presents a heat map of metabolite expression levels, with each column representing a sample and the sample name below. Each row indicates a metabolite, and the color gradient reflects the relative expression level of that metabolite in the sample group. The color gradient corresponds to the numerical values, as shown in the gradient color blocks.

The clustering heat map and VIP bar chart were used to demonstrate the expression patterns of metabolites in the differential groups across samples and their contribution to classification (VIP value). The VIP value measures the intensity of the impact of metabolic expression patterns on sample classification discrimination. A total of 30 major differential metabolites with VIP > 1 were screened in R12 and R20 of Figure 11. Up-regulated metabolites in R12 included: 3-acetylbenzocyanonitrile, 2-(1-benzothiophene-3-yl)acetamide, trehalose, phenyl-1,3-dicarboxylic acid, condurito β-epoxide, ribose, ceramide, 4-[2-(4-cyanophenyl)-1,2-bis(2-ethylamino)ethyl]benzocyanonitrile, edulitol, (E)-octadecen-9-enamide, D-sorbitol, and 3-(4-hydroxyphenyl)prop-2-ene-2-carboxylic acid. Down-regulated metabolites in R20 included: sucrose, fructose, sorbitol, sorbitan, xylose, galactose, 2,8-dimethyldecanoate, phytosterol, 9-octadecenoic acid, 2-methylundecanoate, 3-(4-ethylbenzoyloxy)phenyl]3-methylbenzoate, linalyl camphorol, 3-trifluoromethyl benzylamine, *N*,*N*-dodecyl, eicosapentaenoic acid, arabinitol, raffinose, lignotaric acid, and 9,12-octadecadienic acid (Z,Z).

Among them, sucrose, fructose, xylose, galactose, and raffinose belong to sugar compounds. Sucrose dominates the flavor profile of rice grains, contributing the most sweetness to high-quality japonica rice varieties such as Liaoxing and Daohuaxiang No. 2. Its taste activity value (TAV) > 1 is identified as the primary sweetener [62]. Sucrose not only directly contributes to sweetness but also participates in the starch-sucrose metabolic pathway, positively correlating with gelatinization temperature and indirectly affecting the smoothness of rice texture [63]. Studies have confirmed that significant sucrose reduction under high-temperature or long-term storage conditions leads to diminished rice sweetness and a bland flavor profile. Xylose and galactose, as auxiliary sweeteners (with sweetness approximately 1/5 that of sucrose), primarily influence flavor by modulating the “sweetness perception” and reducing stickiness. Their content decreases with prolonged storage, synchronizing with carbohydrate metabolic disorders and potentially leading to a monotonous flavor profile [64]. Raffinose (with sweetness approximately 22–30% that of sucrose) does not strongly contribute to sweetness but enhances the “softness” of rice and improves texture [65]. Its reduction causes the rice texture to shift from “soft” to “dry and hard,” affecting fullness. 2,8-Dimethyldecanoate, 2-methylundecanoate, and 3-(4-ethylbenzoyl)oxyphenyl]3-methylbenzoate are ester compounds. The fruit aroma contribution of branched fatty acid methyl esters is a crucial component of rice fragrance. 2,8-Dimethyldecanoate and 2-methylundecanoate exhibit strong volatility, imparting fresh fruit aromas such as “apple” and “pear” to rice [66], serving as characteristic aromatic components in high-quality fragrant rice and playing a pivotal role in distinguishing variety-specific aroma profiles [67]. This study confirmed that these ester compounds are prone to volatilization during storage, resulting in a shift from “rich” to “mild” aroma [68]. The structure of 3-(4-ethylbenzoyl)oxyphenyl]3-methylbenzoate is complex and exhibits low volatility, suggesting a potentially limited direct contribution to flavor profile [69].

Phytosterols exhibit potent antioxidant properties, inhibiting lipid oxidation and reducing the formation of “rancid” flavors [70]. Studies indicate that phytosterols influence cell membrane integrity by modulating membrane fluidity, and their increased content helps maintain cellular structural stability under drought stress, indirectly protecting flavor compounds [71]. During storage, the decline in their content weakens lipid antioxidant capacity, accelerating flavor deterioration. Linalool, a key compound that distinguishes fragrant rice varieties, possesses strong “lily” and “rose” aromas as a terpene, serving as the characteristic fragrance for varieties like jasmine and rice flower. It exhibits a high aroma activity value (OAV) and a significant contribution. This study confirms its easy loss during storage, leading to the weakening of rice’s “floral characteristics” and reduced recognizability [72,73].

## 4. Conclusions

This study focused on the fragrance-type japonica rice Liao Xiangjing 1396, and through a 16-month simulated storage experiment combined with metabolomics and metagenomics technologies, systematically elucidated the evolution patterns of key compounds and their biological formation mechanisms during storage. The main conclusions are as follows.

The storage quality of fragrant japonica rice exhibited a phased change characteristic, with fatty acid content increasing from an initial 12.24 mg/g to 17.63 mg/g at 12 months and slightly decreasing to 16.28 mg/g at 16 months. This indicates continuous deterioration in quality during the R20 to R12 stages, while the R11 stage showed stabilization, thereby defining the core research interval for subsequent omics analysis. Metabolomic analysis identified 263 highly credible metabolites, with oxygenated organic compounds (38 types) and lipids/lepidid molecules (24 types) as the core categories, reflecting the essential requirements for energy metabolism maintenance and membrane structure stability during storage. The 12 key differential metabolites (e.g., 4-coumaric acid, citric acid, and palmitic acid) in the R20 and R12 groups involved five major pathways, including amino acid metabolism and lipid metabolism, whose content fluctuations directly influenced the flavor and texture of stored rice. Metagenomic analysis revealed that *Pseudomonas* (60.2%) and Actinobacteria were dominant bacterial phyla, with *Pantoea* spp. (36.17%) and *Pseudomonas* (38.19%) as core potential biomarkers, whose abundance changes effectively characterized the storage stage. In the late storage phase (R12), fungal diversity significantly increased, with the proliferation of Actinobacteria and Bacteroides closely associated with the accumulation of metabolite degradation products. Multiomics association studies confirm that the “fungal community–metabolic pathway–key compound” chain regulation drives quality deterioration: *Klebsiella* spp. dominate the ndhB energy metabolism pathway to accelerate carbohydrate degradation, while multiple genera, including *Pantoea*, collaboratively regulate the mcp chemotaxis pathway to enhance nutrient utilization efficiency. This ultimately leads to reduced flavor compounds such as sucrose and linalool, along with the accumulation of fatty acids and amino acids.

This study systematically elucidated the dynamic association between metabolites and fungal communities during the storage of fragrant japonica rice, providing key targets for quality regulation. Specifically, we have successfully established a correlation between the fluctuations of specific metabolites—such as Free D-Glucose and other key differential compounds—and the aging process in rice grains. While these results provide valuable insights into the biochemical changes affecting quality, further large-scale validation across diverse rice varieties and storage conditions is required to translate these potential biomarkers into a standardized quality assessment tool. Future research should prioritize validating these potential markers under varied storage regimes (e.g., low temperature and modified atmosphere) to assess their universal applicability. Additionally, molecular studies are needed to verify the regulatory mechanisms of key pathways identified herein. Such efforts will facilitate the development of composite preservation technologies and precise early-warning systems, ultimately enhancing the storage stability and commercial value of fragrant japonica rice.

## Figures and Tables

**Table 1:** Differential metabolites and their dominant KEGG pathways.

Metabolite	Regulation	KEGG Pathway Description	Key Pathways
3,4-Dihydroxymandelic Acid	up	Metabolic pathways: tyrosine metabolism	Amino acid metabolism
4-Coumaric acid	down	Metabolic pathways: phenylpropanoid biosynthesis; biosynthesis of secondary metabolites	Flavonoids
9-Octadecenoic acid	down	Cutin, suberine and wax biosynthesis; biosynthesis of unsaturated fatty acids; fatty acid biosynthesis	Metabolism of short-chain fatty acids
Citric Acid	up	Metabolic pathways: glyoxylate and dicarboxylate metabolism; biosynthesis of secondary metabolites; biosynthesis of cofactors; 2-oxocarboxylic acid metabolism; carbon metabolism; biosynthesis of amino acids; alanine, aspartate and glutamate metabolism; citrate cycle (TCA cycle)	Amino acid metabolism; energy metabolism
Free D-Glucose	up	Metabolic pathways: biosynthesis of secondary metabolites; amino sugar and nucleotide sugar metabolism; ABC transporters; galactose metabolism; glycolysis/gluconeogenesis; starch and sucrose metabolism; pentose phosphate pathway	Energy metabolism
L-Asparagine	up	Metabolic pathways; biosynthesis of secondary metabolites;cyanoamino acid metabolism; biosynthesis of amino acids; alanine, aspartate and glutamate metabolism; aminoacyl-tRNA biosynthesis	Amino acid metabolism
L-Glutamic Acid	up	Metabolic pathways: Glyoxylate and dicarboxylate metabolism; arginine biosynthesis; ABC transporters; taurine and hypotaurine metabolism; biosynthesis of cofactors; 2-oxocarboxylic acid metabolism; nitrogen metabolism; carbon metabolism; arginine and proline metabolism; glutathione metabolism; biosynthesis of amino acids; alanine, aspartate and glutamate metabolism; D-amino acid metabolism; aminoacyl-tRNA biosynthesis; histidine metabolism; biosynthesis of secondary metabolites; butanoate metabolism; C5-branched dibasic acid metabolism; porphyrin metabolism	Amino acid metabolism of short-chain fatty acids
Myristic acid	down	Metabolic pathways: fatty acid biosynthesis	Metabolism of short-chain fatty acids
Palmitic Acid	down	Metabolic pathways: fatty acid degradation; fatty acid metabolism; cutin, suberine, and wax biosynthesis; fatty acid biosynthesis; fatty acid elongation; biosynthesis of unsaturated fatty acids	Lipid metabolism of short-chain fatty acids
Ribose	up	Metabolic pathways: pentose phosphate pathway; ABC transporters	Energy metabolism
Stearic Acid	down	Metabolic pathways: biosynthesis of unsaturated fatty acids; fatty acid biosynthesis	Metabolism of short-chain fatty acids

Note: The term “Metabolic pathways” refers to the global KEGG pathway map (map01100), which serves as a reference map containing all known metabolic reactions. Its presence indicates that the identified differential metabolites are integral parts of the general metabolic network. The identification of D-Glucose was based on mass spectral and retention index matching. The stereochemical designation (D-) reflects the biologically relevant isomer in rice, as standard GC-MS without chiral separation cannot resolve L/D enantiomers.

## Data Availability

MetabolomicsData: https://analysis.majorbio.com/metab/overview/task_id/u0pg_2k3ef2ms9qgcceksi7d044 (accessed on 11 February 2026), MetagenomicsData: https://analysis.majorbio.com/metag/overview/task_id/ib2r_9qg9rv3onur6oqe1hncld7 (accessed on 11 February 2026).

## Supplementary Materials

The following supporting information can be downloaded at: https://www.mdpi.com/article/10.3390/metabo16050302/s1, Table S1: List of rice metabolites.
